# Umbilical Cord-Derived Mesenchymal Stem Cells Attenuate S100-Induced Autoimmune Hepatitis via Modulating Th1 and Th17 Cell Responses in Mice

**DOI:** 10.1155/2023/9992207

**Published:** 2023-10-17

**Authors:** Xiaofeng Wei, Xinhong Cheng, Yang Luo, Xun Li

**Affiliations:** ^1^The First Hospital of Lanzhou University, Lanzhou City, Gansu Province, China; ^2^Key Laboratory of Biotherapy and Regenerative Medicine, The First Hospital of Lanzhou University, Lanzhou City, Gansu Province 730000, China; ^3^The First School of Clinical Medicine, Lanzhou University, Lanzhou City, Gansu Province, China

## Abstract

Currently, the first-line treatment for autoimmune hepatitis (AIH) is still the combination of glucocorticoids or immunosuppressants. However, hormone and immunosuppressive therapy can cause serious side effects, such as Cushing syndrome and bone marrow suppression. Previous studies reported on the applicability and safety of mesenchymal stem cells (MSCs) to ameliorate liver inflammation and fibrosis. However, the characteristics of MSCs sources directly contribute to the different conclusions on the mechanisms underlying MSC-mediated immunoregulation. Bone marrow-derived MSCs can exert an immunosuppression effect to ameliorate the S100-induced AIH model by inhibiting several proinflammatory cytokines and upregulating of PD-L1 in liver tissue. It is not clear whether human umbilical cord-derived MSCs (hUC-MSCs) could directly inhibit liver inflammation and ultimately alleviate the dysfunction of hepatocytes in the AIH model. First, hUC-MSCs were extracted from umbilical cord tissue, and the basic biological properties and multilineage differentiation potential were examined. Second, 1 × 10^6^ hUC-MSCs were administered intravenously to AIH mice. At the peak of the disease, serum levels of alanine aminotransferase and aspartate aminotransferase and pathologic damage to liver tissue were measured to evaluate liver function and degree of inflammation. We also observed that the infiltration of CD4^+^ T cells in the liver was significantly reduced. Furthermore, the frequency of the splenic IFN*γ*- and IL-17A- producing CD4^+^ T cells were also significantly decreased, while we only observed an increasing trend in Treg cells in liver tissue. Third, an RNA sequencing analysis of liver tissue was performed, which showed that in the UC-MSC-treated group, the transcriptional profiles of inflammation-related signaling pathways were significantly negatively regulated compared to those of phosphate-buffered saline-treated mice. Collectively, these findings indicated the potential of hUC-MSC to suppress immune responses in immune anomaly mediated liver disease, thus offering a potential clinical option to improve AIH.

## 1. Introduction

Autoimmune hepatitis (AIH) is an autoimmune disease with an unknown pathogenesis and complex etiology, and it can occur in people of all ages and sexes. Serologically, AIH is characterized by elevated transaminase levels, abundant polyclonal immunoglobulin G (IgG), and characteristic autoantibodies [1]. Currently, steroid hormones administered alone or in combination with azathioprine represent the first-line treatment for AIH; however, not all patients respond well to this treatment, with some patients experiencing strong adverse effects [2, 3]. Eventually, a certain percentage of patients with AIH will experience end-stage liver disease and need liver transplantation.

The histopathology of AIH usually presents as interfacial hepatitis via the infiltration of immune cells, such as lymphocytes, macrophages, and plasma cells [4]. In the development of liver inflammation, dendritic, Kupffer, sinusoidal endothelial, and hepatic stellate cells can present antigens and induce an inflammatory cascade [5]. In addition to plasma cells that produce autoantibodies, T cells play an important role in liver inflammation via the production of increased numbers of Th1 and Th17 cells and degradation of regulatory T cell (Treg) activity [6]. T lymphocytes can differentiate into Th1 (secreting TNF-a and interferon (IFN)-*γ*), Th17 (secreting IL-17), Th2 (secreting IL-4 and IL-10), and Tregs (expressing CD25 and Foxp3) according to the environmental cues [7]. Th17 cells, which are characterized by the production of IL-17A, induce immune cell infiltration and mediate hepatic inflammation. Th17 cells are associated with disease pathogenesis in mice and in humans. Tregs are a population of prototypic immunosuppressive T cells that maintain immune tolerance and immune homeostasis. Studies have verified that Treg/Th17 cells maintained a dynamic balance in normal physiological conditions and in disease states [8]. In patients with AIH, defects in the number and function of Tregs are associated with overactive Th1 and Th17 cell responses [9]. S100-induced experimental AIH animal models are appropriate for the study of AIH, which is characterized by infiltration of inflammatory cells in the liver; moreover, the modeling process is short, simple, and reproducible [10].

The liver pathology of AIH indicates that it is mainly an immune-mediated liver disease. Although first-line therapy for AIH has many side effects, this treatment has the most significant positive effects. Therefore, a new therapy that exerts therapeutic effects through immune regulation with fewer side effects and a more lasting therapeutic effect is urgently needed.

Mesenchymal stem cells (MSCs) respond to tissue injury and inflammation by producing anti-inflammatory cytokines and exhibiting chemotactic, anti-inflammatory, and immunomodulatory properties [11]. These cells are involved in various autoimmune diseases, and their safety has been demonstrated through numerous clinical trials of stem cells. The main sources of MSCs are the bone marrow (BM), adipose tissue, umbilical cord blood, and umbilical cord (UC) tissue. Differences in the origin of MSCs have led to different views on the mechanisms and specificity of immune regulation mediated by MSCs. UC-MSCs have the advantages of being more primitive, less immunogenic, easier to obtain, not ethically problematic, and more immunosuppressive compared to MSCs from other sources [12]. Chen et al. [13, 14] reported that the adoptive transfer of bone MSCs attenuated the progression of murine experimental AIH, led to a decrease in serum alanine aminotransferase (ALT) and aspartate aminotransferase (AST) levels, and significantly alleviated lymphocyte infiltration in the liver. However, it is unclear whether human UC-MSCs can be used to treat AIH.

In terms of current clinical applications, human umbilical cord-derived MSCs (hUC-MSCs) are considered to be a better choice of MSCs for clinical applications due to their easier collection and lower immunogenicity than other MSCs [15]. In this study, we established the S100-induced AIH animal model and explored the therapeutic effect and underlying mechanisms of hUC-MSCs in AIH. Our results, for the first time, indicated that transplantation of the hUC-MSCs provides a promising role in controlling the progress of AIH. This study may provide valuable insights into the treatment of human AIH using hUC-MSCs.

## 2. Materials and Methods

### 2.1. Mice

C57BL/6J male mice (6–8 weeks old, 18–22 g) were purchased from the Institute of Veterinary Medicine, Chinese Academy of Agricultural Sciences and raised in a specific pathogen-free animal room of the Medical College of Lanzhou University. The animal room alternated between day and night conditions, and the indoor temperature was 24°C. All mice used in this study were 6–8 weeks old and treated according to National Institutes of Health guidelines for the use of experimental animals. All protocols were approved by the Ethics Committee of the First Hospital of Lanzhou University (approval no: LDYYLL2022-421, the ethical approval has been uploaded as an attachment).

### 2.2. AIH Model Induction and hUC-MSC Treatment in AIH

#### 2.2.1. S100 Protein Preparation

Proteins were extracted and used immediately. First, the livers from C57BL/6J mice were perfused with phosphate-buffered saline (PBS) until they turned white, and the tissue was then removed, cut into pieces, and processed into a suspension using a tissue processor (Tissulyser-48, Shanghai Jinxing, China). The suspension was stored at −80°C and then thawed to 24°C three times to fully disrupt the cells. It was then centrifuged at 150×g for 15 min, and the supernatant was collected and placed in an ultracentrifuge (Optima XPN-100, Beckman Coulter, USA) and centrifuged at 4°C for 1.5 hr at 100,000×g. The resulting supernatant represented the S100 protein and was used for immunization [10].

#### 2.2.2. AIH Model

An AIH model was developed as previously described [10]. Briefly, the freshly extracted liver S100 protein was separated by passing it through a 90 cm CL-6b Sepharose column (Pharmacia, Freiburg). Induction of AIH was performed via intraperitoneal injection of S100 protein (2.5 mg/200 *µ*L) that was fully emulsified in an equal volume of complete Freund's adjuvant (CFA; Sigma, St. Louis, MO, USA) on day 0, and a repeat injection was performed on day 7 after modeling. All 24 Mice were randomly divided into three experimental groups: control group, AIH model group (mice were injected with 200 *µ*L PBS via the tail vein on days 5 and 21), and UC-MSC-treated group (AIH model mice were injected with 1 × 10^6^ UC-MSCs via the tail vein on days 5 and 21).

### 2.3. Detection of Serum Chemical Levels

The serum was isolated from the blood samples of each mouse by 3,000 rpm centrifugation for 15 min. Then, according to the protocol, the serum contents of ALT, AST, and globulin (GLB) were detected using an automatic biochemistry analyzer (AU5800, Beckman, USA).

### 2.4. Liver Histological Examination

For the general histopathology evaluation, the paraffin-embedded liver samples were sectioned (5 *μ*m), stained with hematoxylin and eosin, and placed under a microscope (Olympus LX73, Japan) for analysis at different magnifications. Pathological sections from each animal were examined by two experienced liver pathologists who were blinded to the study objectives, and the degree of liver inflammation was assessed according to Knodell's histological activity index (HAI) score system [16]. The HAI represented the combined score for necrosis, inflammation, and fibrosis of the liver.

### 2.5. Antibodies and Flow Cytometry

Murine antibodies (Abs) against anti-CD4-percp5.5, anti-IL17A-PE, anti-IFN*γ*-FITC, anti-CD25-PE, and anti-Foxp3-FITC (all from eBioscience, San Diego, CA, USA) were used for flow cytometry (FCM) according to the manufacturer's instructions. For intracellular staining of cytokines, 250 *µ*L cell systems were stimulated in vitro with 1 *µ*L of PMA (12.5 mg/mL), 1 *µ*L of lonomycin (0.25 mg/mL), 1 *µ*L of brefeldin A (0.75 mg/mL), and 1 *µ*L of monesin (0.35 mg/mL) (Multi Sciences, Hangzhou, China) for 5 hr before the fluorescence-assisted cell sorting (FACS) analysis. Finally, the cells were resuspended in 500 *µ*L of PBS and analyzed via FCM (BD FACS, Canto, II, USA).

### 2.6. Liver Immunofluorescence

After deparaffinization, antigen retrieval, and blocking, paraffin-embedded liver sections were incubated with antimouse CD4 mAb (GB13064-2, Servicebio, China) overnight at 4°C in a wet chamber. Next, goat antimouse HRP-labeled secondary antibody (GB23301, Servicebio, China) and cy3-TSA were added, and the nuclei were counterstained with DAPI. The slides were then washed and prepared for observation.

### 2.7. MSC Preparation

#### 2.7.1. Isolation

UC tissue was obtained from the discarded samples of healthy pregnant women who delivered at the First Hospital of Lanzhou University. MSCs were extracted and purified from the human UC using the tissue block adherence method. After 7 days, spindle cells could be observed crawling out from the edge of the tissue block, which was recorded as the P0 generation. The medium was replaced every 3 days until cell confluence reached 80%–90%, and the cells were then digested and passaged (Trypsin, Biological Industries, Israel). Each passage of digestion increased the passage of cells by one, which were labeled P1, P2, P3, and so forth. All cells were cultured in a medium (DMEM-F12) containing 10% fetal bovine serum (Gibco) and placed in a 5% CO_2_ incubator at 37°C.

#### 2.7.2. Identification

The MSCs (3 × 10^6^) were resuspended in 600 *µ*L of PBS and then were divided into five 12 × 75 mm polystyrene tubes: control tube; tube 1 (PE mouse antihuman CD73), tube 2 (percp mouse antihuman CD90), tube 3 (APC mouse antihuman cd105), tube 4 (FITC mouse antihuman CD44), tube 5 (Positive cocktail and FITC negative cocktail), tube 6 (positive isotype control cocktail and FITC mouse IgG1 isotype control). The corresponding antibodies (Human MSC Analysis Kit, Sino Biological, China) were added to the tubes, which were then incubated at 24°C in the dark for 30 min. Next, the cells were washed twice with 500 *µ*L of PBS. Finally, the cells were resuspended in 500 *µ*L of PBS per tube and analyzed immediately on a flow cytometer (Agilent Novocyte Advanteon Dx, USA). The MSCs used in the experiment were from passages 4 to 5.

#### 2.7.3. Adipogenic and Osteogenic Differentiation

The cells were plated on a six-well plate, and after almost complete fusion, the corresponding osteogenic induction solution (hUC-MSC osteogenic differentiation complete medium, HUXUC-90021, Cyagen, China) and adipogenic induction solution (hUC-MSC adipogenic differentiation complete medium, HUXUC-90031, Cyagen, China) were added. The medium was changed according to the operating procedure. After approximately 4 weeks, Alizarin red staining and oil red O staining were performed, and the cells were photographed and observed under a microscope (Olympus LX73, Japan).

### 2.8. Tissue Homogenization and RNA Extraction

Total RNA was extracted from mouse liver tissue using TRIzol (Ambion) and then further purified through two phenol-chloroform treatments. The residual DNA was removed by adding RQ1 DNase (Promega, Madison, WI, USA). The purified RNA was resuspended in enzyme-free water. The isolated RNA was assessed for quality and quantity using a NanoDrop One (Thermo), and the absorbance of the sample was measured at 260/280 nm. The RNA integrity was further verified through 1% agarose gel electrophoresis. Liver tissue specimens with RNA of insufficient quantity, poor quality, and loss of integrity were eliminated.

### 2.9. RNA Sequencing and Analysis

Library construction and sequencing were completed by the Ruixing Science and Technology Company, Wuhan. Samples were prepared by capturing mRNA (Vazyme, N401), removing DNA using RQ1 (Promega), and preparing a directed RNA sequencing (RNA-Seq) library through stranded mRNA-Seq (KK8544). mRNA was converted to double-stranded DNA and ligated to an adaptor (KK8726, Roche). A library was successfully constructed by amplifying, purifying, and quantifying the interrupted DNA to 300–500 bp. The library preparations were finally subjected to 150 nt paired-end sequencing on an Illumina NovaSeq 6000 system. Strands labeled with dUTP (second-strand cDNA) were not amplified and were thus were available for strand-specific sequencing.

### 2.10. Statistical Analysis

Statistical differences among the three groups were analyzed through one-way analysis of variance with GraphPad Prism 8 software. All values are expressed as the mean ± standard deviation, and values were considered statistically significant at *P* < 0.05.

## 3. Results

### 3.1. Biological Characteristics of hUC-MSCs

**Figure 1:**
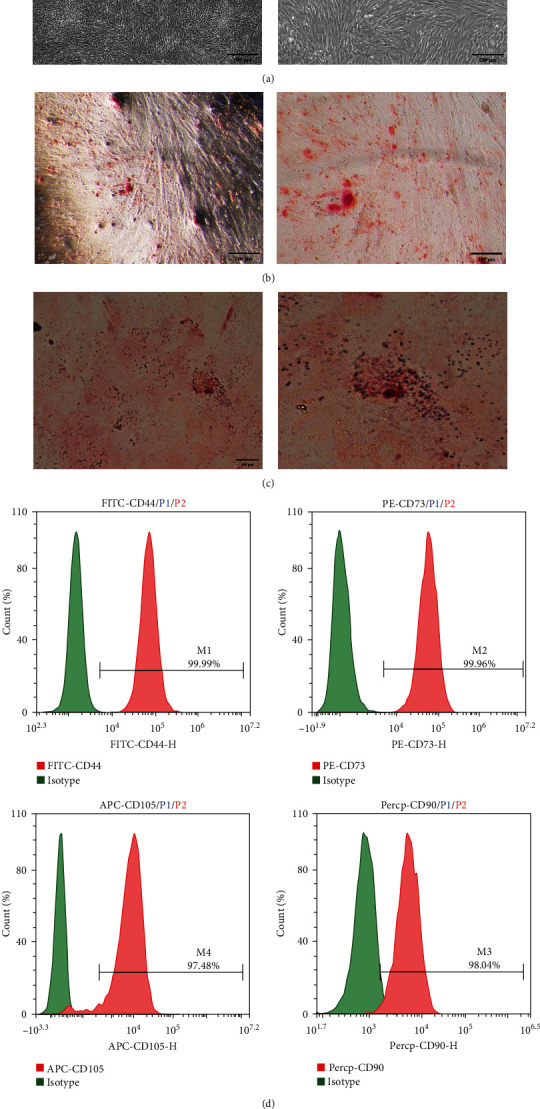
Minimal criteria of human umbilical cord-derived mesenchymal stem cells (hUC-MSCs). (a) Microscopic observation of the hUC-MSCs morphology, (b) osteogenesis differentiation by staining with Alizarin red, (c) adipogenesis differentiation by staining with oil red O, and (d) cell surface markers of hUC-MSCs were measured by flow cytometry (CD73, CD90, CD105, and CD44).

hUC-MSCs were obtained from fresh healthy UCs. To determine the characteristics of hUC-MSCs, we examined their phenotype, morphology, and growth patterns. First, we observed that the isolated adherent cells exhibited a spindle-shaped fibroblast-like morphology during cell culture and passage (Figures 1(a) and S1). Second, these cells had the ability to differentiate into osteoblasts (Figure 1(b)) and adipocytes (Figure 1(c)) after culture in osteogenic and adipogenic media followed by Alizarin red and oil red-O staining respectively. Third, the FCM results indicated that the surface markers of hUC-MSCs CD44 (99.99%), CD73 (99.96%), CD105 (97.48%), and CD90 (98.04%), were positive (Figure 1(d)), and the levels of hematopoietic markers, such as CD45, HLA-DR, CD14, CD19, and CD34, were all negative (Figure S2). These results suggested that the isolated hUC-MSCs retained the features of MSCs. Collectively, these results demonstrated that the hUC-MSCs meet the criteria of MSCs as defined by the International Society for Cellular Therapy (ISCT) [17] and could thus be used in subsequent experiments.

### 3.2. hUC-MSCs Can Effectively Ameliorate S100-Induced Hepatitis

**Figure 2:**
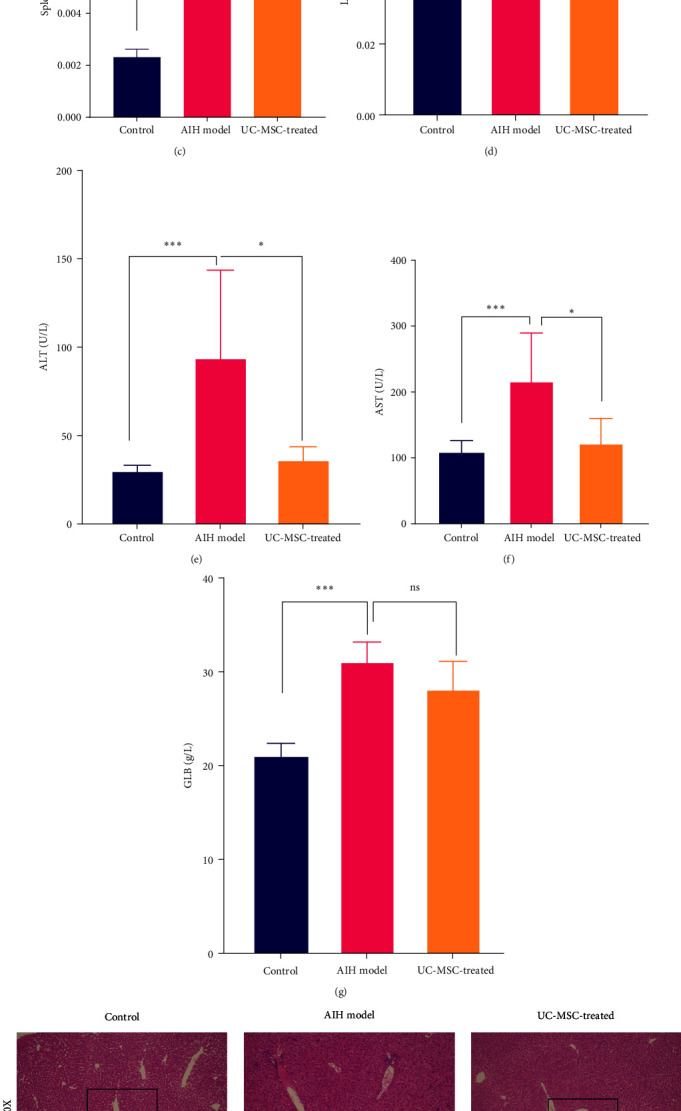
Human UC-MSCs alleviate S100-induced experimental AIH in mice. (a) Intervention and treatment schedule to establish the AIH model, mice were sacrificed on day 35 after the first immunization, (b) representative general morphology of livers and spleens from the different groups are shown on day 35 (*n* = 8), (c and d) changes in spleen and liver indexes on day 35 (*n* = 8) ( ^*∗*^*P* < 0.05,  ^*∗*^ ^*∗*^ ^*∗*^*P* < 0.01), (e–g) changes in blood serum alanine aminotransferase (ALT), aspartate aminotransferase (AST), and globulin (GLB) levels in mice (*n* = 8) ( ^*∗*^*P* < 0.05,  ^*∗*^ ^*∗*^ ^*∗*^*P* < 0.01), and (h and i) representative images of liver tissues in each group (hematoxylin and eosin staining) and Knodell-HAI scores of mouse liver (*n* = 8) ( ^*∗*^ ^*∗*^ ^*∗*^*P* < 0.01). HAI, histological activity index.

To explore the role of hUC-MSCs in experimental AIH, we used the liver injury model induced using S100 protein as the AIH model [10]. The protocol in Figure 2(a) shows the time points of the S100 and hUC-MSCs treatments. The emulsifier was mixed with S100 protein and CFA and injected intraperitoneally into mice on days 0 and 7, 1 × 10^6^ hUC-MSCs were injected on days 5 and 21 through the tail vein, and the mice were euthanized on day 35.

We then observed the effect of MSCs based on the gross liver and spleen samples, serological indexes, and liver pathological changes. Overall, the liver and spleen of mice in the UC-MSC-treated group decreased to a certain degree than the AIH model group (Figure 2(b)). We also found that the liver and spleen indexes of mice in the AIH model group were higher than those of the control group (liver index: 0.040 ± 0.005 vs. 0.069 ± 0.005, *P* < 0.01; spleen index: 0.0023 ± 0.000 vs. 0.0073 ± 0.001, *P* < 0.05). However, those of the UC-MSC-treated group were significantly lower than those of the AIH group (liver index: 0.069 ± 0.005 vs. 0.049 ± 0.006, *P* < 0.01. spleen index: 0.0073 ± 0.001 vs. 0.005 ± 0.002, *P* < 0.05) (Figures 2(c) and 2(d)). Subsequently, the serum levels of ALT, AST, and GLB, which represent liver function changes in serology, were evaluated. As shown in Figure 2(e)–2(g), the serum levels of ALT, AST, and GLB were all elevated in the AIH model group compared with the control group (ALT: 30.236 ± 1.546 vs. 94.816 ± 43.567, *P* < 0.01. AST: 108.544 ± 15.766 vs. 217.488 ± 64.555, *P* < 0.01. GLB: 21.130 ± 1.158 vs. 31.223 ± 3.581, *P* < 0.01). After injecting the hUC-MSCs, the ALT and AST levels in the UC-MSC-treated group decreased compared with that of the AIH model group. Finally, the liver pathology was identified using HE staining, which revealed the liver structure. As expected, the liver structure of control mice was normal while the liver lesions in the AIH model group were characterized by polymorphonuclear leukocyte infiltration. Furthermore, a number of lymphocytes were confined to the portal area, the lobules were destroyed, and interfacial hepatitis was observed, thus indicating the typical histological characteristics of AIH. However, after the administration of hUC-MSCs, the symptoms of hepatitis were significantly reduced. As shown in the preserved lobular structure, focal necrosis was not observed, and the infiltration of lymphocytes was reduced (Figure 2(h)). Moreover, the Knodell-HAI score (Figure 2(i)) of the UC-MSC-treated group was significantly lower than that of the AIH model group (4.000 ± 2.236 vs. 8.875 ± 2.027, *P* < 0.01). These results showed that the AIH model was successfully established and adoptive transplantation of UC-MSC had a noticeable therapeutic effect on AIH.

### 3.3. hUC-MSCs Inhibit Aberrant Th1 and Th17 Responses in S100-Induced AIH

**Figure 3:**
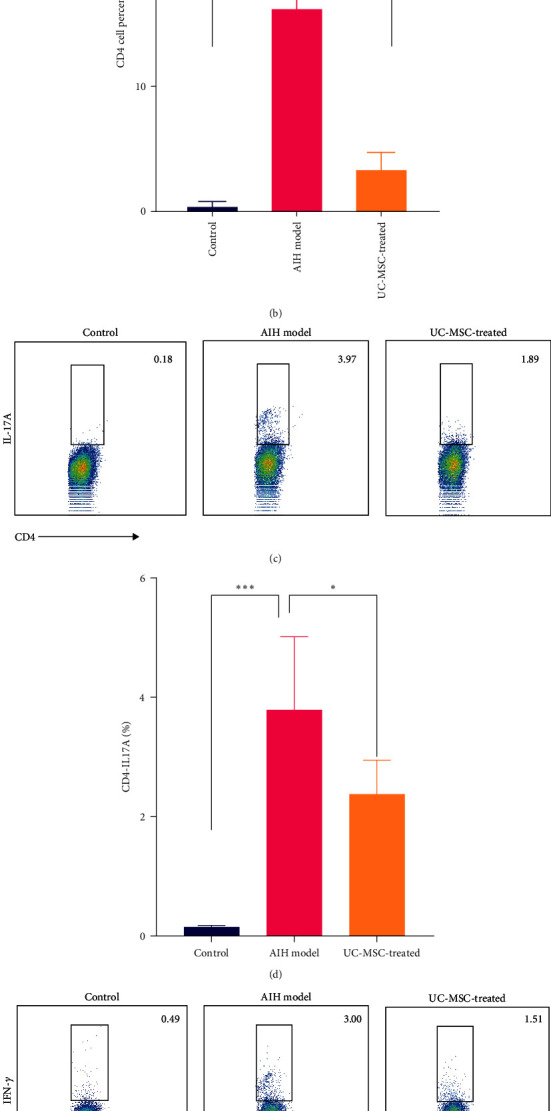
Human UC-MSCs prevent CD4^+^ T cells from infiltrating the liver and reduce splenic Th1 and Th17 cell differentiation. (a and b) Mice were sacrificed on day 35 after the first immunization. Immunofluorescence staining showing CD4^+^ T cell infiltration in liver sections from each group. Quantitative analysis of fluorescence imaging using ImageJ software (*n* = 5) ( ^*∗*^*P* < 0.05,  ^*∗*^ ^*∗*^ ^*∗*^*P* < 0.01), (c and d) splenic Th17 cells (CD4^+^ IL-17A^+^) examined using FACS for each group (*n* = 5) ( ^*∗*^*P* < 0.05,  ^*∗*^ ^*∗*^ ^*∗*^*P* < 0.01), and (e and f) splenic Th1 cells (CD4^+^ IFN-*γ*^+^) examined using FACS for each group (*n* = 5) ( ^*∗*^*P* < 0.05,  ^*∗*^ ^*∗*^ ^*∗*^*P* < 0.01).

In the livers of control mice, T-cell infiltration was not observed. However, in the livers of mice injected with S100, considerable CD4^+^ T-cell infiltration was observed. As expected, the percentage of and total number of CD4^+^ T cells in the liver of the UC-MSC-treated group were decreased to a greater extent compared with those in the AIH model group (3.533 ± 0.988 vs. 14.400 ± 6.923, *P* < 0.05) (Figures 3(a) and 3(b)). This finding indicates that hUC-MSCs substantially suppressed the migration of CD4^+^ pathogenic T cells to the liver. Next, we determined whether the hUC-MSC treatment influenced the percentage of Th17 and Th1 cells. The FCM results showed that the number of splenic CD4^+^ IL-17A^+^ T cells and CD4^+^ IFN-*γ*^+^ T cells were notably higher in the AIH model mice while the number of Th17 (Figures 3(c) and 3(d)) and Th1 cells (Figures 3(e) and 3(f)) were markedly lower in the UC-MSC-treated mice. Moreover, after UC-MSC transplantation, no significant increase was detected in Treg cells in the spleen in UC-MSC-treated mice compared with that AIH model group (Figure S3). Thus, the ability of hUC-MSCs to mediate immunoregulatory functions in vivo may contribute to their therapeutic effect on AIH.

### 3.4. Potential Mechanisms Underlying the hUC-MSCs Treatment Effects in the AIH Model

**Figure 4:**
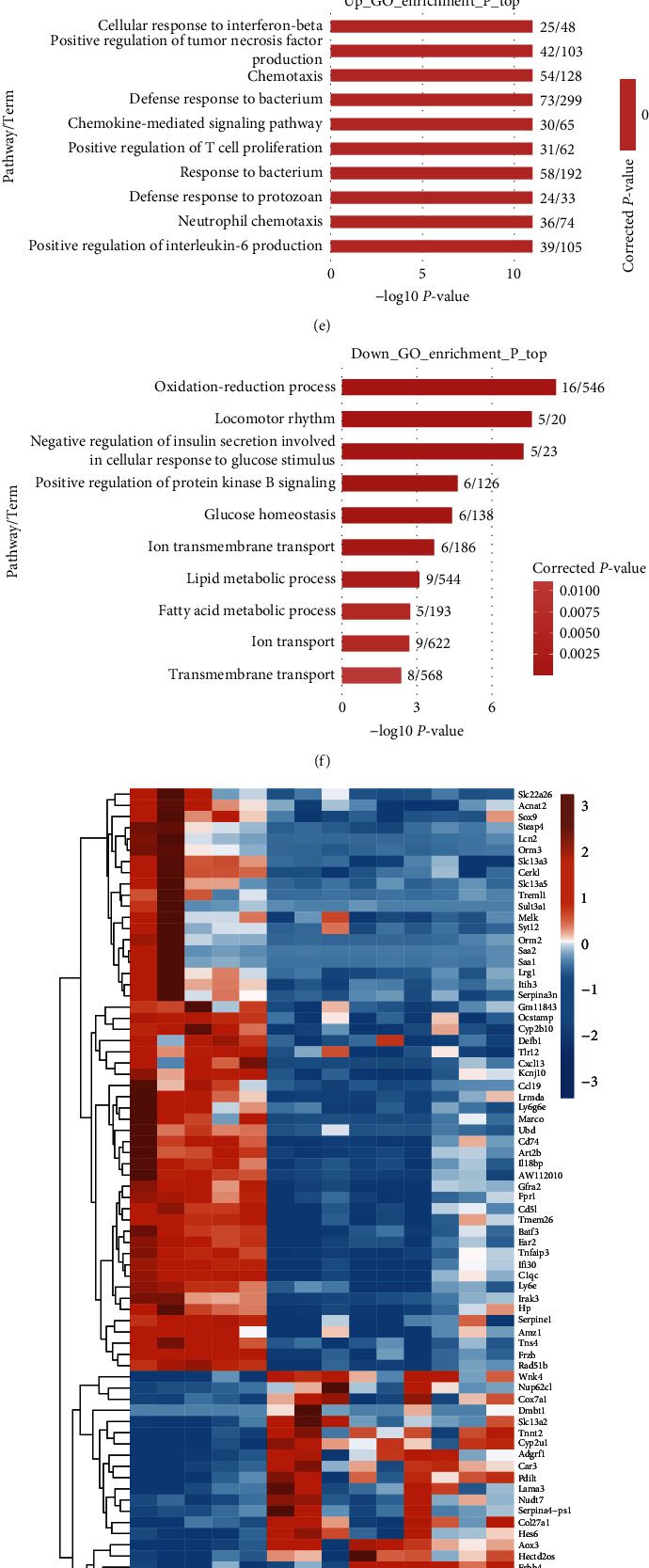
RNA-Seq analysis of gene expression and signaling pathway changes in AIH liver tissue. (a) Cluster analysis based on sample correlation coefficient (samples with higher correlation coefficients have higher similarity and closer clustering relationships), (b) heatmap clustering of all DEGs (horizontal is the sample, vertical is the gene), (c) volcanic map of DEGs between control group and AIH model group, (d) volcanic map of DEGs between model group and UC-MSC-treated group (the horizontal axis in the figure represents the multiple changes in expression level (log value is taken as the base of 2), while the vertical axis represents the statistical significance of the differences in expression level changes (log value is taken as the base of 10)), (e and f) GO analysis of up-regulated and down-regulated genes between control and AIH model group, (g) heatmap of DEGs among the three groups, and (h) analysis of differential immune cells among the three groups by CIBERSORTx.

To further explore the mechanisms underlying hUC-MSCs therapy, we performed RNA sequencing of the liver tissues harvested on day 35 postcellular treatment to explore the possible therapeutic mechanisms. Five liver tissue samples were obtained from each group. All sequencing data were subjected to strict quality control measures and normalization before analysis. First, a cluster analysis was performed according to the sample correlation coefficient; One liver sample of a hUC-MSC-treated mouse was an outlier and was therefore discarded (Figures 4(a) and 4(b)). This study defines differentially expressed genes (DEGs) based on fold change greater than or equal to 2 or less than or equal to 0.5, with a correction of *P* less than 0.05. Compared with the control group, the AIH model group had 1,633 upregulated genes and 224 downregulated genes (Figure 4(c)).

To better explain the potential role of DEGs, we performed Gene Ontology (GO) analyses. The 1,633 upregulated genes were mainly enriched in the following GO terms: cellular response to interferon-beta, positive regulation of tumor necrosis factor production, chemokine-mediated signaling pathway, positive regulation of T-cell proliferation, neutrophil chemotaxis, and positive regulation of interleukin-6 production (Figure 4(e)). By contrast, the downregulated genes were mainly associated with the following GO terms: oxidation–reduction process, lipid metabolic process, and fatty acid metabolic process (Figure 4(f)).

Fewer DEGs were observed between the UC-MSC-treated group and the AIH model group (Figure 4(d)). Heatmap analysis of the DEGs demonstrated that significant differences occurred between the groups. Compared with the model group, 51 genes related to inflammation response and immunity were significantly downregulated in the UC-MSC-treated group, including Cd5l, Tnfaip3, Serpina3n, Cxcl13, Defb1, Fpr1, Tlr12, HP, CCL19, Saa1, CD74, and C1qc, whereas more than 30 genes were upregulated after the hUC-MSC treatment, including 10 genes involved in the oxidation–reduction process (Aox3, Nr1d1, mt-Co2, mt-Cytb, mt-Nd4l, Mup11, Cox7a1, Cyp2u1, Mup16, and Mup12) and 20 genes involved in the developmental process (Cdh4, Pcsk4, Pdilt, Cspg5, Col27a1, Gpr110, Nr1d1, Nudt7, Dbp, mt-Co2, mt-Cytb, Dmbt1, Hes6, Lama3, Mup11, Wnk4, Mup16, Tnnt2, Mup12, and Erbb4) (Figure 4(g)). Thus, after hUC-MSC treatment, genes related to inflammation and immune responses were downregulated, while genes related to development were significantly upregulated compared with those in the AIH model group. To further explore the changes of different immune cells, we used CIBERSORTx software (https://cibersortx.stanford.edu/runcibersortx.php) to estimate the cellular composition based on gene expression (Figure 4(h)). This information can be used as a reference for subsequent studies.

## 4. Discussion

Epidemiological studies show that the incidence rate of AIH is increasing year by year [18]. Although most patients with AIH are women, this disease also affects men and impacts all races and ages around the world [19, 20]. For the treatment of AIH, the use of glucocorticoids alone or in combination with immunosuppressants is considered the most effective first-line treatment method [19]; however, the side effects of long-term hormone and immunosuppressive therapy, such as Cushing's syndrome and BM suppression, require intermediate treatment [21]. The currently developed second- and third-line therapies also have various shortcomings. Some patients with an insufficient response may progress to cirrhosis [22], and the only currently viable treatment option for advanced cirrhosis is liver transplantation [23]. However, patients with AIH who require transplantation may experience long waiting lists, and AIH recurs in 8%–12% and 36%–60% of patients who receive liver transplants within 1 and 5 years, respectively [19]. These disappointing therapeutic outcomes coupled with the increasing prevalence necessitate the development of alternative and effective therapies. Therefore, we attempted to explore whether MSCs can be developed into a new therapeutic strategy for improving AIH.

MSCs are a type of pluripotent nonhematopoietic stem cell with immune regulatory ability [11], and they are widely used to treat various inflammatory diseases [24, 25]. Moreover, MSCs have been reported to exert therapeutic effects in various animal models of autoimmune diseases, such as lupus erythematosus and rheumatoid arthritis [26]. The immune regulatory ability of MSCs is plastic, and the high content of inflammatory factors in the body promotes their immunosuppressive effects. When the inflammatory factors in the body are insufficient, MSCs can also enhance the immune response of the body. These characteristics have led researchers to believe that MSCs can help maintain immune homeostasis in the body by improving the immune imbalance [27].

The pathophysiology of AIH is relatively complex, and immune dysfunction is one of the causes [28]. The loss of tolerance of the liver to autoantigens is considered a potential pathogenic mechanism of AIH, and the activation of autoimmune T cells is a key step in the pathophysiology of AIH [29]. Based on the pathogenesis of AIH and the immune regulatory effects of stem cells, we speculate that MSCs can exert immune regulation and restore the immune homeostasis of AIH. At present, limited research has focuses on stem cell therapies for AIH. Chen et al. [13] demonstrated that in the S100-induced mouse AIH model, tail vein administration of bone marrow-derived MSCs (BMSCs) significantly reduced liver damage. They also showed that BMSCs transplantation can upregulate LMO7 expression by inhibiting the TGF-*β* pathways, thereby limiting hepatic fibrogenesis to help alleviate AIH [14]. Compared to BM-derived stem cells, UC-derived stem cells are more primitive, can be obtained using noninvasive methods, have lower immunogenicity, and have more advantages. However, their role in AIH has yet to be studied. To our knowledge, this study shows for the first time that infusing hUC-MSCs into AIH mice can suppress liver inflammation and improve liver function based on the results revealing the strong inhibitory ability of hUC-MSCs.

This study mainly demonstrated that hUC-MSCs can alleviate AIH inflammation in mouse mice. The stem cells used here were derived from discarded UC tissue from the first hospital of Lanzhou University, and MSCs were intermediately extracted using the tissue block attachment method. The cells were identified through FCM and differentiation experiments, and they met the minimum standards specified by the ISCT and showed high purity and multidirectional differentiation ability. This identification method is internationally recommended and recognized [17]. Many types of AIH animal models have been established, and the most commonly used are constructed using S100 [10] and concanavalin A [30]. The AIH model constructed by the Losher team using S100 protein showed a slower development compared with the concanavalin A model and thus is more in line with the characteristics of most AIH patients; moreover, the modeling process is simple and economical [31]. Although AIH is more likely to occur in women, male C57BL/C6 mice have the strongest susceptibility to AIH based on previous work on multiple mouse models, with such susceptibility observed in almost all of the established models [10]. Previous AIH models constructed using S100 also mostly used male C57BL/6J [32, 33].

In this study, experimental AIH was induced in the liver of C57BL/6J male mice by intraperitoneal injection of the S100 antigen on days 0 and 7. Two weeks after induction, mice developed liver inflammation, which peaked at week 4. Pathological analysis showed that the S100-induced AIH model exhibited inflammatory immune cell infiltration and local liver cell necrosis. In addition to the pathology, the ALT and AST levels are the most sensitive indicators of liver dysfunction [20]. In the S100-induced AIH model, we demonstrated that hUC-MSCs significantly reduced ALT and AST levels. In addition, hUC-MSCs significantly reduced the infiltration of monocytes into the lobular center or portal vein region in hUC-MSC-treated mice compared with that in the model or control group.

The specific type of cell infiltration that is reduced in the liver and the role of CD4^+^ T cells in AIH have been confirmed through different mouse models. The number of infiltrating T cells in the liver is related to the degree of inflammation [34–36]. Multiple studies have shown that Th17 cells can lead to the deterioration of liver function in S100-induced AIH models or patients with AIH [37–39]. High frequencies of Th17 cells in the blood and liver and high IL-17 levels in the serum are associated with poor prognosis in AIH [38]. Research has also shown that the Th1-derived proinflammatory cytokines are crucial for the development of AIH in mouse models [40, 41].

To evaluate the effect of hUC-MSCs on CD4^+^ T cell subsets, this study detected changes in CD4^+^ T cells in the liver tissue mouse of AIH mice using immunofluorescence methods. The spleen, as a peripheral immune organ, has been shown to generate invasive T cells. After splenectomy, liver-infiltrating T cells are significantly reduced and less fibrous tissue is observed in the spleen, which facilitates the production of a large number of immune cells. The immune status of the liver can be determined by detecting changes in spleen immune cells [42]. Cellular changes in Th1, Th17, and Treg cells in splenic tissue were detected by FCM. The results showed that except for a decrease in the number of CD4^+^ T cells infiltrating the liver of mice treated with hUC-MSCs, the proliferation of Th1 and Th17 was significantly inhibited in the periphery. These results provide sufficient evidence that hUC-MSCs can alleviate liver inflammation and improve liver function by inhibiting CD4^+^ T proliferation and peripheral CD4^+^ T cells migration to the liver.

To further elucidate the mechanism underlying the improvement in AIH after tail vein injection of hUC-MSCs, transcriptome sequencing was performed on five mouse liver tissues from each group in this study. After hUC-MSCs therapy, inflammatory genes such as Saa1, Cxcl13, and CD74 were downregulated. This study also analyzed the changes in the immune cell population using CIBERSORTx online analysis tools. Mononuclear macrophages and natural killer cells showed significant changes among the three groups, which may play an important role in the immune response. Professor Shi's team has demonstrated that MSCs cultured under hypoxia can secrete IGF2 factor, which can preprogram developing macrophages to obtain anti-inflammatory properties associated with oxidative phosphorylation, thereby effectively improving autoimmune encephalomyelitis [43]. The hUC-MSCs obtained in this study can also exert anti-inflammatory effects without undergoing hypoxic cultivation. However, the underlying mechanisms remain to be revealed.

Although our data indicated a role of hUC-MSCs in experimental AIH, the study presented several limitations that may have affected the results. First, It's possible that the hUC-MSCs can affect other cell types, such as endothelial cells, Kupffer cells, and monocytes/macrophages. In addition, previous studies indicated that MSC-exosomes can transfer miRNA to alleviate experimental AIH. Thus, further studies are needed to clarify the type of immune cells or secreted exosomes that specifically target the liver tissue. Second, because the complicated immune microenvironment can affect the heterogeneity of MSCs, thereby contributing to changes of their migration, proliferation, and immunosuppression, whether different MSC subsets possess the same range of immunomodulatory function should be verified. Third, a limited time of observation was implemented in our study. Therefore, the long-term adverse effects of treatment on AIH remain to be investigated [44].

In summary, the results of this study indicate that tail vein injection of hUC-MSCs can significantly alleviate AIH in mice, thus providing new evidentiary support for the further clinical application of UC-MSCs. RNA sequencing of liver tissue was performed to preliminarily explore the possible molecular and cellular mechanisms. Future studies can focus on the fate and outcomes of MSCs in vivo, the specific mechanisms by which MSCs inhibit CD4^+^ T cells, and the relationship between stem cells and other cells.

## 5. Conclusions

To the best of our knowledge, We have revealed for the first time that hUC-MSCs can considerably attenuate liver inflammation and AIH pathology in an S100-immunized experimental AIH model. These results were dependent on the inhibition of splenic Th1 and Th17 cell responses and decreases in the frequency of liver-infiltrating CD4^+^ T cells. Furthermore, we also observed clear changes in gene expression patterns relative to the functional characteristics and capacity of the AIH hepatocytes. These findings support the notion that selecting more effective source-derived MSCs may represent an optimal strategy for stem cell-based therapies for AIH.

## Data Availability

The data used to support the findings of this study are available from the corresponding author upon request.

## Supplementary Materials

Supplementary 1 Figure S1: morphology of mesenchymal stem cells of different generations. Figure S2: identification of MSCs surface markers by flow cytometry. Figure S3: splenic Treg cells examined using flow cytometry for each group (*n* = 5).

Supplementary 2 Approval by Ethics Committee of LZU No.1 hospital.
