# Supplementary material for: Umbilical Cord-Derived Mesenchymal Stem Cells Attenuate S100-Induced Autoimmune Hepatitis via Modulating Th1 and Th17 Cell Responses in Mice
Source: Stem Cells Int. 2023 Oct 17;2023:9992207. doi: 10.1155/2023/9992207 (PMC10597736; doi:10.1155/2023/9992207)
Supplement: Supplementary 1 — Figure S1: morphology of mesenchymal stem cells of different generations. Figure S2: identification of MSCs surface markers by flow cytometry. Figure S3: splenic Treg cells examined using flow cytometry for each group (n = 5). [file 9992207.f1.docx]

**Supplement 1**

**Figure S1.**


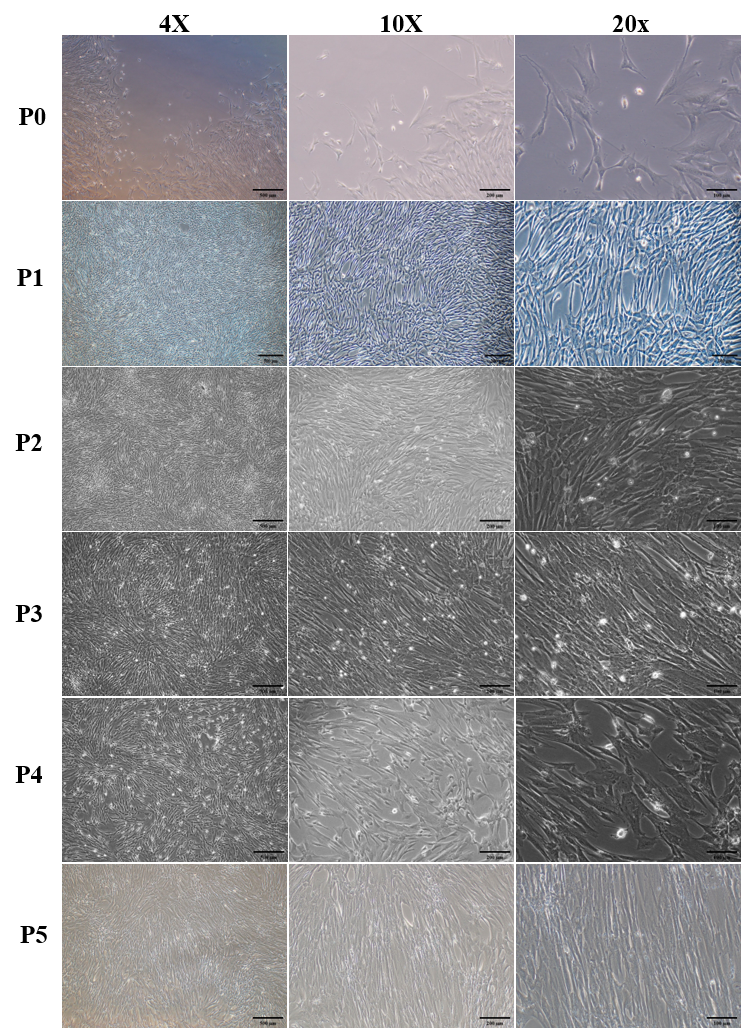


**Figure S1. Morphology of mesenchymal stem cells of different generations at three magnifications under a microscope.**

**Figure S2.**

**Figure S2. Identification of MSCs surface markers by flow cytometry**
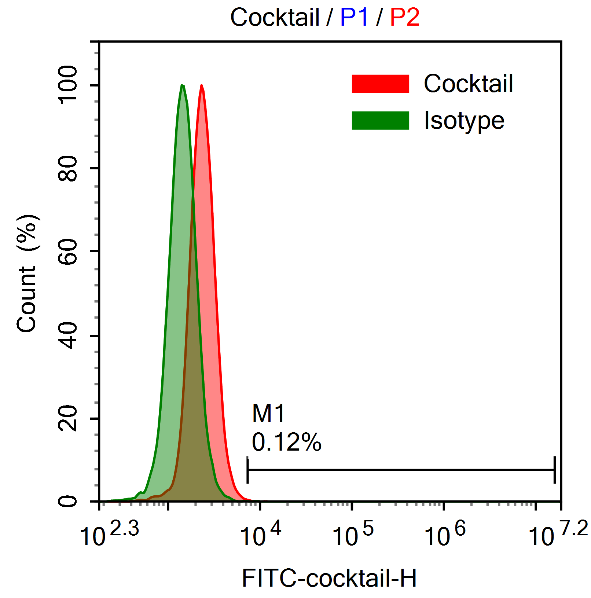
**.**

The FITC-cocktail includes: FITC-CD45，FITC-CD34，FITC-CD14, FITC-CD19，FITC-HLA-DR

Isotype：FITC Mouse IgG1 isotype control

**Figure S3.**


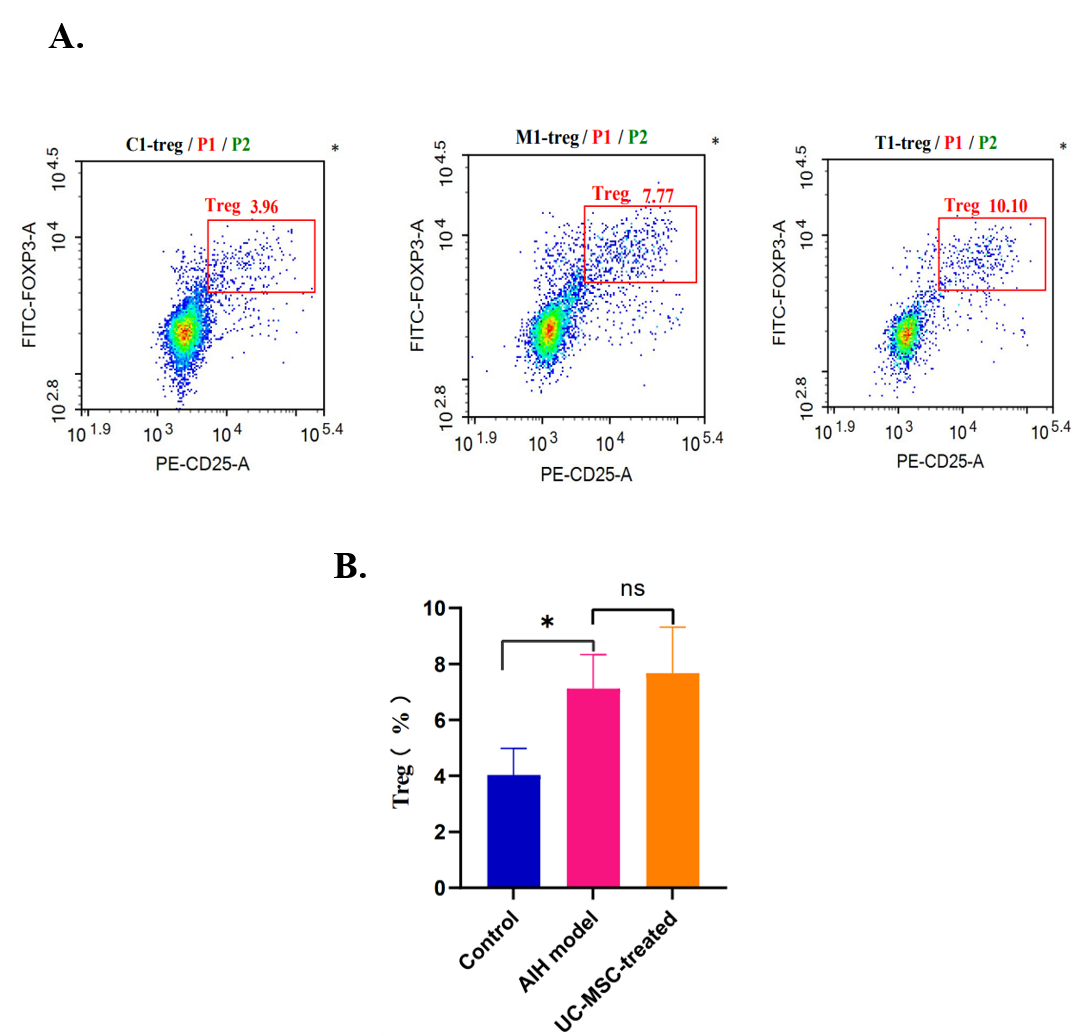


**Figure S3. Splenic Treg cells (CD4+CD25+Foxp3+) examined using FACS for each group (n=5) (*P<0.05).**
