# Supplementary material for: Umbilical Cord-Derived Mesenchymal Stem Cells Attenuate S100-Induced Autoimmune Hepatitis via Modulating Th1 and Th17 Cell Responses in Mice
Source: Stem Cells Int. 2023 Oct 17;2023:9992207. doi: 10.1155/2023/9992207 (PMC10597736; doi:10.1155/2023/9992207)
Supplement: Supplementary 2 — Approval by Ethics Committee of LZU No.1 hospital. [file 9992207.f2.pdf]

Approval by Ethics Committee of LZU No. 1 Hospital

Approval No.:LDYYLL-2022-421	Date of review:30/09/2022	
Name of research plan: Umbilical Cord derived Mesenchymal Stem Cells ameliorate Experimental Autoimmune Hepatitis by inhibiting CD4+ T cell Migration and Th17 Differentiation		
Applicant/trial product/SFDA No.:<br>The No. 1 Hospital of Lanzhou University	Type	Scientific research
Principal Investigator/organization: Xiaofeng Wei / The No. 1 Hospital of Lanzhou University		
Document (with version number)<br>Research plan ☑ Have ☐No<br>Waiver of Informed consent form ☑ Have ☐No<br>Application for ethical review of animal experiments ☑ Have ☐No		
Results of Ethics Committee's review:<br>1. The Committee reviewed the technical plan and informed consent form for the participants, decisions for the program are as follows :<br>☑ Approve<br>☐Approve after amendment<br>☐ Disapprove<br>☐Termination or suspension<br>2. Will the progress of the research reviewed by the ethics committee continually?<br>☑ Yes ☐No<br>3. The frequency of review is once every 12 months from the date of approval, the ethics committee has right to change the frequency of continued review based on actual progress.<br><br>LZU No. 1 hospital Ethics Committee (Seal):<br><br>Date: 30/09/2022		
